# Influenza Virus Surveillance in Pakistan during 2008-2011

**DOI:** 10.1371/journal.pone.0079959

**Published:** 2013-11-08

**Authors:** Nazish Badar, Uzma Bashir Aamir, Muhammad Rashid Mehmood, Nadia Nisar, Muhammad Masroor Alam, Birjees Mazhar Kazi, Syed Sohail Zahoor Zaidi

**Affiliations:** Department of Virology, National Institute of Health, Chak Shahzad, Islamabad, Pakistan; National Institutes of Health, United States of America

## Abstract

**Background:**

There is little information about influenza among the Pakistani population. In order to assess the trends of Influenza-like-Illness (ILI) and to monitor the predominant circulating strains of influenza viruses, a country-wide lab-based surveillance system for ILI and Severe Acute Respiratory Illness (SARI) with weekly sampling and reporting was established in 2008. This system was necessary for early detection of emerging novel influenza subtypes and timely response for influenza prevention and control.

**Methods:**

Five sentinel sites at tertiary care hospitals across Pakistan collected epidemiological data and respiratory samples from Influenza-like illness (ILI) and severe acute respiratory illness (SARI) cases from January 2008 to December 2011. Samples were typed and sub-typed by Real-Time RT-PCR assay.

**Results:**

A total of 6258 specimens were analyzed; influenza virus was detected in 1489 (24%) samples, including 1066 (72%) Influenza type A and 423 (28%) influenza type B viruses. Amongst influenza A viruses, 25 (2%) were seasonal A/H1N1, 169 (16%) were A/H3N2 and 872 (82 %) were A(H1N1)pdm09. Influenza B virus circulation was detected throughout the year along with few cases of seasonal A/H1N1 virus during late winter and spring. Influenza A/H3N2 virus circulation was mainly observed during summer months (August-October).

**Conclusions:**

The findings of this study emphasize the need for continuous and comprehensive influenza surveillance. Prospective data from multiple years is needed to predict seasonal trends for vaccine development and to further fortify pandemic preparedness.

## Introduction

Influenza is an acute viral and highly contagious respiratory infection causing significant morbidity and mortality worldwide with public health implications [1,2]. Two main types (A and B) of influenza virus are responsible for recurrent epidemics in humans. As compared to influenza B, Influenza A has a more significant impact on public health due to its faster evolution and diverse host range [3,4]. Seasonal Influenza viruses continually circulate in yearly epidemics (mainly during the winter months in temperate climates), while antigenically novel strains emerge sporadically as pandemic viruses [5]. The recent emergence of a reassortant pandemic H1N1 virus in April 2009 and its subsequent global spread within weeks embodied the pandemic threat forecast by scientists for years. 

Influenza exhibits a distinct seasonal pattern in temperate areas with marked peaks in the winter (typically December–April in the Northern Hemisphere and June–September in the Southern Hemisphere) [2,6]. Tropical and subtropical regions with mild winters also have seasonal variations in disease incidence, sometimes linked to rainy season [7]. Moreover, the seasonal pattern is generally less pronounced in tropical and sub-tropical areas [2,6]. Therefore it is essential to understand the seasonal trends of various influenza subtypes for enhancing preparedness against seasonal as well as pandemic influenza. 

Global influenza surveillance has been conducted through the WHO Global Influenza Surveillance and Response System (GISRS) since 1952. This network monitors evolution of influenza viruses and provides recommendations on areas including laboratory diagnostics, vaccines, antiviral susceptibility and risk assessment. It also serves as a global alert mechanism for the emergence of influenza viruses with pandemic potential. The network currently comprises six WHO Collaborating Centers, four Essential Regulatory Laboratories and 136 institutions in 106 WHO Member States, which are recognized by WHO as National Influenza Centers [1,8,9].

Pakistan is a South Asian country with tropical to temperate climate and a population of over 180 million. Since 1980, sporadic influenza surveillance has been conducted under the National Influenza Center (NIC) at the National Institute of Health, Islamabad. However in 2008 a sentinel lab-based influenza surveillance network was established in collaboration with the US-Centers for Disease Control and Prevention (CDC). The objective was to identify circulating influenza virus strains, characterize clinical manifestations of influenza and identify vulnerable population groups. The present study reports surveillance and laboratory data from January, 2008 to December, 2011.

## Materials and Methods

### Case Definition for Sample Collection

WHO prescribed case definitions for influenza-like illness (ILI) and severe acute respiratory infection (SARI) were used for screening outpatient and hospitalized patients: ILI cases were defined as those with sudden onset of fever (>38°C) and cough/sore throat within seven days of onset, while patients with sudden onset of fever (>38°C), cough/sore throat that required hospital admission within 7 days were termed as SARI [10].

### Study Population

Coordinated activities in collaboration with all provinces were carried out at the national influenza center (NIC) located in NIH, Islamabad. The sentinel site for sample collection for NIC, Islamabad is Federal Govt. Services Hospital. There are four more provincial sentinel sites at Hayatabad Medical Complex Hospital in Khyber Pakhtunkhwa, King Edward Medical University Hospital in Punjab, Civil Hospital in Sindh and Bolan Medical Complex Hospital in Baluchistan province. These hospitals were designated the sub centers for influenza surveillance. At least 5–10 nasopharyngeal samples were collected from patients meeting the ILI or SARI case definition each week. Samples received from case contacts or patients with history suggestive of influenza were also processed. 

### Ethical Clearance

The surveillance and sampling protocols were approved by Pakistan National Institute of Health Internal Review Board. A formal consent written or verbal was obtained from each subject; however, the patient identities have not been disclosed at any stage. The institutional board was informed of the specific needs with reference to study setting and approved this mode of consent. A check box was included in the data form to document the consent taking procedure.

### Laboratory Diagnosis

Throat and/or nasopharyngeal swabs collected from suspected cases in 2-3 ml viral transport medium (Virocult®) were stored at -70°C before use. RNA was extracted from samples using Qiagen QIAmp Viral RNA mini kit and eluted in 60ul elution buffer. The samples were analyzed by one-step real-time reverse transcription-polymerase chain reaction (rRT-PCR) on Applied Biosystems platform ABI 7500 following recommended US-CDC protocol [11]. The assay was performed using AgPath-IDTM One –Step RT-PCR (Ambion; California, USA). Briefly, 25ul PCR mixture containing 0.5ul each of probe (FAM labeled), forward and reverse primers, 1ul enzyme mix, 12.5 ul of 2X master mix, 5 ul nuclease-free water and 5ul of extracted RNA was subjected for amplification with conditions; reverse transcription at 50°C for 30 min, Taq inhibitor activation 95°C for 10 min and 45 cycles at 95°C for 15s, extension step of 55°C for 31s. 

### Statistical Analysis

The influenza patients were divided into four groups on the basis of detection of A/H1N1, A/H3N2, A(H1N1)pdm09 and influenza B respectively, for comparison of age groups and clinical presentation. The epidemiological and laboratory data were analyzed using a Pearson’s chi-squared test to compare epidemiological factors (age groups, clinical presentation and ILI/SARI). *P* values were considered significant if found < 0.05. Statistical analysis was done using SPSS 16 software.

## Results

**Figure 1:**
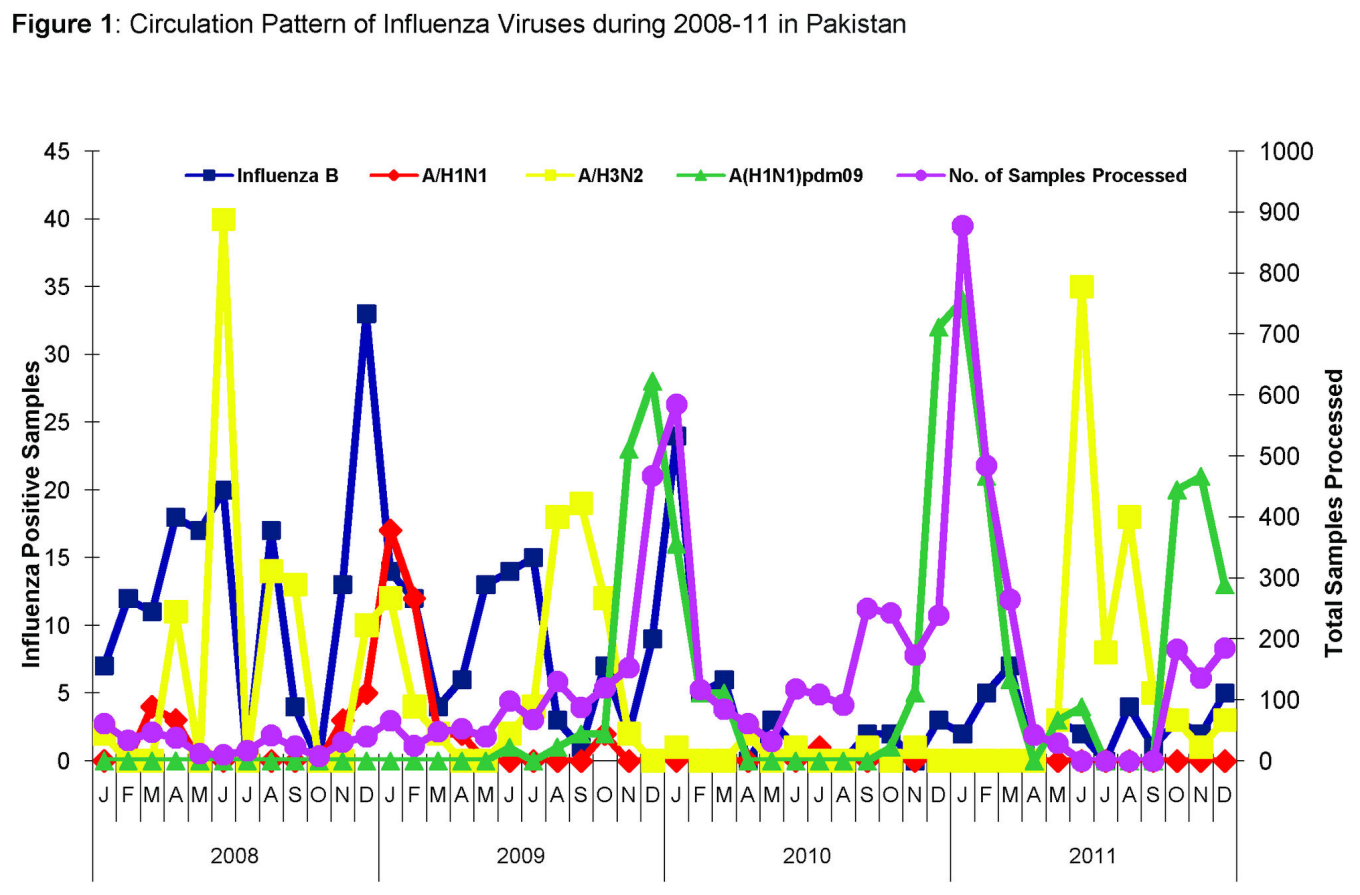
Circulation Pattern of Influenza Viruses during 2008-11 in Pakistan. Monthly/Yearly distribution of different Influenza subtypes during 2008-11.

During 2008-2011, 6258 specimens were tested for influenza by real-time RT-PCR. Out of these 1489 (24%) were found positive for influenza viruses (Table 1). 1066 (72%) were found positive for Influenza A and 423 (28%) for influenza B. Sub-typing of Influenza A revealed that 872 (82%) were A/(H1N1)pdm09, 169 (16%) as A/ H3N2 and 25 (2%) seasonal A/H1N1 . A low level of influenza circulation was observed in 2008 (77/364; 21%) but showed a marked increase in 2009 (367/1357; 27%), due to emergence of novel Influenza A/(H1N1)pdm09 (Table 1). In early 2010, influenza detection rate was only 18% (372/2105), but a second wave of high activity was observed in November-December (Figure 1). Influenza positivity showed an upsurge again in 2011 (673/2432; 27%). Throughout the study period a baseline circulation of Influenza B was observed (Figure 1). 

**Table 1:** Year wise distribution of Influenza Subtypes (2008–2011).

**Year**	**Total No. of Samples Tested**	**Total Positive Samples** (**n, %**)	**Samples Positive for Influenza A or B**		**Samples Positive for Influenza A subtypes**		
			**Influenza B (n, %)**	**Influenza A (n, %)**	**A/H1N1 (n, %)**	**A/H3N2 (n, %)**	**A(H1N1)pdm09 (n, %)**
2008	364	77 (21%)	49 (64%)	28 (36%)	6 (21%)	22 (79%)	NA
2009	1357	367 (27%)	102 (28%)	265 (72%)	18 (7%)	74 (28%)	173 (65%)
2010	2105	372 (18%)	169 (45%)	203 (55%)	1 (0.50%)	9 (4%)	193 (95%)
2011	2432	673 (28%)	103 (15%)	570 (85%)	-	64 (11%)	506 (89%)
**Total**	**6258**	**1489 (24%)**	**423 (28%)**	**1066 (72%)**	**25 (2%)**	**169 (16%)**	**872 (82%)**

During 2008-11, samples tested positive for influenza in the various regions in the following order; Federal Capital: 53%, Punjab: 24%, Sindh: 13%, Khyber Pakhtunkhwa (KPK): 8%, and Baluchistan: 2%. Although influenza viruses were detected year-round, the types/subtypes varied remarkably. A/H3N2, A (H1N1) pdm09) and influenza B virus was detected in both ILI and SARI cases whereas A/H1N1 was detected mainly from ILI cases. Year round Influenza B circulation was observed in Federal Capital and Punjab with infrequent activity in Sindh, Baluchistan and KPK during winter (Dec-Feb). Pandemic Influenza A/(H1N1) pdm09 was first reported in June, 2009 in Punjab and remained in circulation up to December, 2011 across the country. 

**Figure 2:**
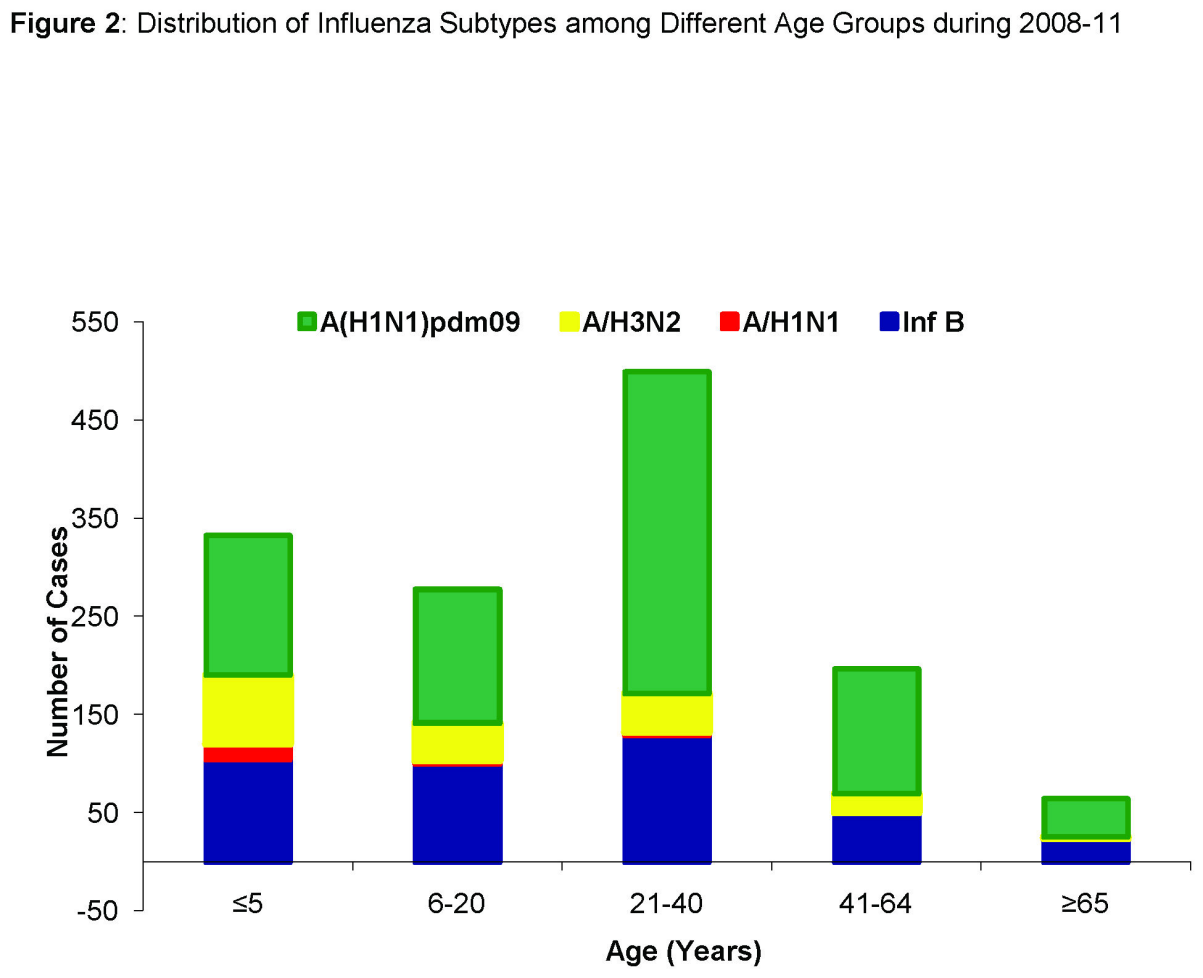
Distribution of Influenza Subtypes among Different Age Groups during 2008-11. Pattern of influenza viruses detection by subtypes in different age groups. Influenza B is shown in Dark blue, A/H1N1 in Red, A /H3N2 in Yellow and A/H1N1 pdm 09 in Green. Note: Cases with missing age data were not included.

The mean ages for infection by different influenza subtypes i.e. seasonal A/H1N1, A/H3N2, A/(H1N1)pdm09 and influenza B were 10 ±14 years, 17 ±17 years, 27 ±18.7 years and 23 ± 20 years respectively. Age was found to be a significant factor among influenza virus subtypes by analysis of variance (*p*<0.001). Analysis of data included from the pre-pandemic period (January, 2008 to May, 2009) (n=596) revealed that 21-40 years and 41-64 years age groups had the highest influenza positivity rates (23-27%). Analysis of intra- and post-pandemic periods showed similar influenza positivity rates for these age groups (Figure 2); 21-40 years (483/1797; 27%) and 41-64 years (189/779; 24%) with A/(H1N1)pdm09 positivity of 66% {21-40years; 328/ 500} and 64% {41-64years;127/197} amongst them (Table 2). Data analysis showed that the 21-40 years age group and 41-60 years were most affected during the pre-pandemic, pandemic and post pandemic periods

**Table 2:** Distribution of seasonal and Influenza A(H1N1)pdm09 viruses among different age categories.

**Age Categories (yr**)	**Influenza A subtypes (n=1076)**					
					**Negative**	
				**Influenza B**	**(n=4759)**	***p*-value**
	**A/H1N1**	**A/H3N2**	**A(H1N1) pdm09**	**(n=423)**		
	**(n=25)**	**(n=179)**	**(n=872**)			
≤5	16	70	142	105	1419	0.001*
6-20	4	38	136	100	1595	0.015*
21-40	4	39	328	129	976	0.045*
41-64	1	19	127	50	420	0.189
≥65	0	1	39	25	37	0.708
**Total**	**25**	**167**	**772**	**409**	**4447**	

**Figure 3:**
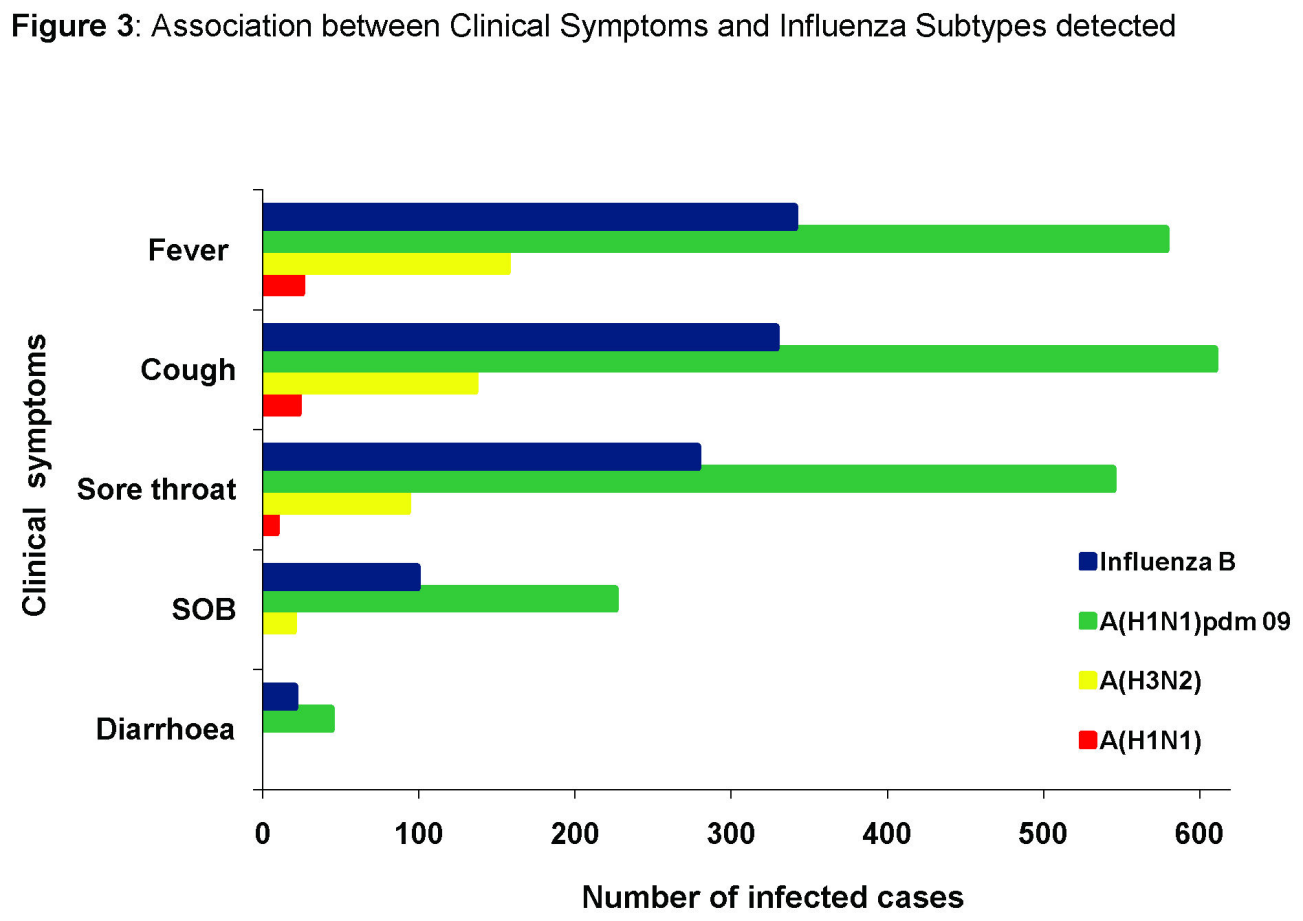
Association between Clinical Symptoms and Influenza Subtypes detected. Comparison of clinical symptom like fever, cough, sore throat and shortness of breath with influenza types and subtypes.

Univariate analyses of clinical data showed that fever, cough and sore throat were significantly higher in ILI patients positive for all influenza subtypes (Figure 3). However, children ≤5 years and adults between 21-40 years positive for A/H1N1 and A/H3N2 influenza were more often presented with fever and cough than those patients who tested positive for A(H1N1)pdm09 and influenza B. Sore throat was more commonly associated with infection caused by A(H1N1)pdm09 (81%) and Influenza B (61%) in children of 0-5 years of age. Diarrhea was a significant clinical feature only in children under 5 years of age infected with A (H1N1) pdm09. There were 1157 (78%) ILI and 332 (22%) SARI patients with laboratory confirmed influenza virus infections. Influenza A (H1N1)pdm09 was the most common subtype identified among both the ILI (55 %) and SARI (72 %) patients followed by Influenza B virus seen in (30 %) ILI and (23%) SARI cases respectively (Table 3).

**Table 3:** Clinical Symptoms in patients with Influenza-Like Illness (ILI) and Severe Acute Respiratory Illness (SARI).

**Clinical Symptoms**	**Influenza Positive n (%)**		**Influenza Negative n (%)**		**Total Cases**	***p*-value**
	**ILI**	**SARI**	**ILI**	**SARI**		
Fever	879 (87)	223 (65)	3834 (90)	333 (66)	5269	0.001*
Cough	854 (80)	244 (81)	3508 (86)	405 (80)	5011	0.001*
Sore Throat	732 (55)	194 (54)	3616 (89)	415 (82)	4957	0.002*
Shortness of Breath	176 (14)	169 (47)	2296 (67)	216 (43)	2857	0.001*
Diarrhea	34 (3)	35 (12)	29 (2.3)	10 (2)	108	*0.932*

Comparison between Influenza positive and negative cases among non-hospitalized (ILI) and hospitalized (SARI) on basis of clinical symptoms (except diarrhea) using Chi-square test. *p* values *of* <0.05 (indicated with *) were considered significant.

## Discussion

Importance of influenza as an emerging and re-emerging disease has been increasingly recognized over the last three decades due to the appearance of novel and potentially pandemic strains of avian and swine origins. The brunt of these influenza pandemics is substantial in terms of morbidity, mortality and economic cost and there is potential for serious social disruption. In view of Pakistan’s diverse geographical terrain and associated climatic changes, continued surveillance is essential for analyzing disease trends and its burden on the health system Improved understanding of influenza viruses’ epidemiology and their seasonal trends through laboratory based surveillance is indispensable for proficient and effective channeling of limited resources towards pandemic preparedness. 

Data obtained from this study confirmed that influenza viruses were prevalent across Pakistan, and affected all age groups during 2008-2011. All influenza types/subtypes including A/H1N1, A/H3N2, Influenza B and the novel A/(H1N1)pdm09 were detected. Seasonal influenza viruses remained in circulation during winter from November, 2008 to March, 2010 with a peak in December while in summer, sporadic influenza cases were detected. This pattern is comparable to tropical countries such as Northern Vietnam, Thailand and Singapore where influenza viruses circulate year-round, and unlike temperate regions where transmission occurs with marked seasonality [12,13]. However, seasonal influenza variation has been linked to changes in environmental factors such as solar radiation, humidity and temperature [13-15]. As a country located in the temperate zone, these factors might arguably effect influenza circulation in Pakistan. Furthermore, yearly variation in influenza detection rates has been well-documented for temperate climates [14,15]. Our subtype data reflects influenza circulation trends similar to other temperate climates such as in Turkey and Northern China, which have documented high influenza transmission rates during winter [16,17]. We therefore conclude that influenza circulation in Pakistan showed an overlapping pattern of temperate and tropical regions during the study period, even though it is geographically placed in temperate region. This can help local and regional public-health officials to improve pandemic preparedness through regular monitoring of influenza-related illness and death rates, investigation of unusual respiratory disease outbreaks, and characterization of current influenza viruses.

During the first half of 2008-2011, the dominant Influenza A subtype was A/H3N2 while A(H1N1)pdm09 was detected from June, 2009 onwards; this pattern of virus circulation was similar to Europe [18] where higher clinical consultation rates were observed for A(H1N1)pdm09 in 2009. Researchers from China, Bangladesh and Guatemala have reported circulation of A/H3N2 viruses during summer and trend similar to observations from our study [19–21]. Due to emergence of a novel virus during study period, only inferences/corollaries can be made regarding exact peak influenza transmission period and presence or absence of a distinct seasonal trend.

Overall, both influenza A and B viruses co-circulated through the surveillance period. Influenza Seasonal A/H1N1 and A/H3N2 viruses, however, dominated in different periods with little overlap, i.e. Seasonal A/H1N1 showed circulation for a brief period in 2008, with a marked increase in the first half of 2009, while A/H3N2 was detected as predominant influenza A subtype prior to appearance of A (H1N1) pdm09. This observation is similar to studies from Delhi (India) [22] where A/H3N2 positive cases surged before the pandemic. From June to October 2009, sporadic cases of pandemic influenza A/(H1N1) pdm09 were seen, but the peak activity began in November-December 2009, possibly due to colder weather, when influenza viruses transmit more easily [23,24] Later distinct peaks of A/ (H1N1) pdm09 activity were observed in winter seasons of 2010 and 2011. These findings correspond to status in other regions where A (H1N1) pdm09 either completely replaced seasonal influenza [25–27] or co-circulated with flu viruses.

 We observed that Influenza B circulated throughout the year, and detection rates were comparable to influenza A viruses. Simmerman et. al., in Thailand also found that Influenza B virus infections did not demonstrate a consistent/regular seasonal pattern and virus was detected throughout the year [28]. Although less pronounced than Influenza A and with little evidence of any pandemic potential, influenza B still has a noteworthy impact on morbidity and hospitalization rates, as reported by Esposito et. al., in Italy [29] who showed that children infected with influenza B had significantly higher hospitalization rates than cases infected with Influenza A(H1N1) pdm09. In our study, Influenza B followed pdmH1N1 as a close second in both ILI and SARI cases when compared to other influenza A subtypes. Therefore, the effect of year-round presence of influenza B viruses on the health care system must be estimated alongside analysis of the influenza A associated morbidity and mortality. 

The fact that a novel pandemic virus was co-circulating in the predicted influenza season offered an opportunity for comparison of epidemiology, clinical presentation and outcome for seasonal versus pandemic viruses. We know pandemic viruses characteristically infect younger age groups and healthy adults in comparison to seasonal influenza where extremes of age and individuals with co-morbid conditions are the susceptible population groups [30]. In contrast, pandemic viruses such as A(H1N1) pdm09 target young adult population from 21-40 years in age. Our study showed detection of A/H1N1 and A/H3N2 was higher in children less than 10 years of age than A/(H1N1)pdm09 and Influenza B which reflects the observations of Bin Cao et. al., who demonstrated higher susceptibility rates for A/H3N2 and A/H1N1 in children below 10 years of age [19]. There is limited available data that shows that other respiratory viruses such as respiratory syncytial virus (RSV), human metapneumovirus and adenoviruses co-circulate in influenza season causing ILI and SARI infections and could partly explain lower positivity rates in children for pandemic H1N1 (Personal communications/unpublished data). Serological surveillance in the United Kingdom during the pandemic has shown higher positivity and hospitalization rates in 15-44 years age group with low reported rates for children [30]. In the United States, the elderly age group (>65yrs) were less severely affected by the pandemic virus [31] which is similar to our results with only 14% elderly patients being affected by A/(H1N1)pdm09. 

## Conclusions

We believe that emergence of a pandemic virus in early years of influenza surveillance in Pakistan has highlighted both the strengths and limitations of the existing system and provides an opportunity for improvement. Continuous monitoring of seasonal influenza subtypes in both ILI and SARI cases can help determine seasonal trends and ultimately help assess the true disease burden. Prospective data of multiple years through multicentre influenza surveillance will help to predict seasonality and fortify pandemic preparedness initiatives. Furthermore it will also help to better define seasonal patterns in the circulation of influenza A and B viruses, regional differences in influenza seasonality, as well as to determine optimal periods to implement influenza vaccination programs among priority populations.
